# Atorvastatin and Aspirin as Adjuvant Therapy in Patients with SARS-CoV-2 Infection: A structured summary of a study protocol for a randomised controlled trial

**DOI:** 10.1186/s13063-020-04840-y

**Published:** 2020-10-30

**Authors:** Nirmal Ghati, Ambuj Roy, Sushma Bhatnagar, Sumit Bhati, Sudha Bhushan, Manjit Mahendran, Abhishek Thakur, Pawan Tiwari, Tanima Dwivedi, Kalaivani Mani, Ritu Gupta, Anant Mohan, Rakesh Garg, Anita Saxena, Randeep Guleria, Siddharthan Deepti

**Affiliations:** 1 Department of Cardiology, All India Institute of Medical Sciences (AIIMS), Ansari Nagar East, New Delhi, 110029 India; 2 Department of Onco-Anaesthesia, Dr. B.R.A Institute-Rotary Cancer Hospital, All India Institute of Medical Sciences (AIIMS), New Delhi, India; 3 Department of Pulmonary Medicine and Sleep Disorders, All India Institute of Medical Sciences (AIIMS), New Delhi, India; 4 Department of Laboratory Medicine, National Cancer Institute (Jhajjar, Haryana), All India Institute of Medical Sciences (AIIMS), New Delhi, India; 5 Department of Biostatistics, All India Institute of Medical Sciences (AIIMS), New Delhi, India; 6 Department of Laboratory Oncology, Dr. B.R.A Institute-Rotary Cancer Hospital, All India Institute of Medical Sciences (AIIMS), New Delhi, India

**Keywords:** COVID-19, Randomised control trial, Protocol, Statin, Aspirin, Mortality

## Abstract

**Objectives:**

To assess the impact of adding statin (atorvastatin) and/or aspirin on clinical deterioration in patients infected with SARS-CoV-2 who require hospitalisation. The safety of these drugs in COVID-19 patients will also be evaluated.

**Trial design:**

This is a single-centre, prospective, four-arm parallel design, open-label, randomized control trial.

**Participants:**

The study will be conducted at National Cancer Institute (NCI), Jhajjar, Haryana, which is a part of All India Institute of Medical Sciences (AIIMS), New Delhi, and has been converted into a dedicated COVID-19 management centre since the outbreak of the pandemic. All RT-PCR confirmed cases of SARS-CoV-2 infection with age ≥ 40 years and < 75 years requiring hospital admission (patients with WHO clinical improvement ordinal score 3 to 5) will be included in the trial. Written informed consent will be taken for all recruited patients. Patients with a critical illness (WHO clinical improvement ordinal score > 5), documented significant liver disease/dysfunction (aspartate transaminase [AST] / alanine aminotransferase [ALT] > 240), myopathy and rhabdomyolysis (creatine phosphokinase [CPK] > 5x normal), allergy or intolerance to statins or aspirin, prior statin or aspirin use within 30 days, history of active gastrointestinal bleeding in past three months, coagulopathy, thrombocytopenia (platelet count < 100000/ dl), pregnancy, active breastfeeding, or inability to take oral or nasogastric medications will be excluded. Patients refusing to give written consent and taking drugs that are known to have a significant drug interaction with statin or aspirin [including cyclosporine, HIV protease inhibitors, hepatitis C protease inhibitor, telaprevir, fibric acid derivatives (gemfibrozil), niacin, azole antifungals (itraconazole, ketoconazole), clarithromycin and colchicine] will also be excluded from the trial.

**Intervention and comparator:**

In this study, the benefit and safety of atorvastatin (statin) and/or aspirin as adjuvant therapy will be compared with the control group receiving usual care for management of COVID-19. Atorvastatin will be prescribed as 40 mg oral tablets once daily for ten days or until discharge, whichever is earlier. The dose of aspirin will be 75 mg once daily for ten days or until discharge, whichever is earlier. All other therapies will be administered according to the institute’s COVID-19 treatment protocol and the treating physician’s clinical judgment.

**Main outcomes:**

All study participants will be prospectively followed up for ten days or until hospital discharge, whichever is longer for outcomes. The primary outcome will be clinical deterioration characterized by progression to WHO clinical improvement ordinal score ≥ 6 (i.e., endotracheal intubation, non-invasive mechanical ventilation, pressor agents, renal replacement therapy, ECMO requirement, and mortality). The secondary outcomes will be change in serum inflammatory markers (C-reactive protein and Interleukin-6), Troponin I, and creatine phosphokinase (CPK) from time zero to 5th day of study enrolment or 7th day after symptom onset, whichever is later. Other clinical outcomes that will be assessed include progression to Acute Respiratory Distress Syndrome (ARDS), shock, ICU admission, length of ICU admission, length of hospital admission, and in-hospital mortality. Adverse drug effects like myalgia, myopathy, rhabdomyolysis, hepatotoxicity, and bleeding will also be examined in the trial to assess the safety of the interventions.

**Randomisation:**

The study will use a four-arm parallel-group design. A computer-generated permuted block randomization with mixed block size will be used to randomize the participants in a 1:1:1:1 ratio to group A (atorvastatin with conventional therapy), group B (aspirin with conventional therapy), group C (aspirin + atorvastatin with conventional therapy), and group D (control; only conventional therapy).

**Blinding (masking):**

The study will be an open-label trial.

**Numbers to be randomised (sample size):**

As there is no existing study that has evaluated the role of aspirin and atorvastatin in COVID-19 patients, formal sample size calculation has not been done. Patients satisfying the inclusion and exclusion criteria will be recruited during six months of study period. Once the first 200 patients are included in each arm (i.e., total 800 patients), the final sample size calculation will be done on the basis of the interim analysis of the collected data.

**Trial Status:**

The institutional ethical committee has approved the study protocol (*Protocol version 3.0* [June 2020]).

Participant recruitment starting date: 28^th^ July 2020

Participant recruitment ending date: 27^th^ January 2021

Trial duration: 6 months

**Trial registration:**

The trial has been prospectively registered in ***Clinical Trial Registry – India*** (ICMR- NIMS): Reference no. CTRI/2020/07/026791 (registered on 25 July 2020)].

**Full protocol:**

The full protocol is attached as an additional file, accessible from the Trials website (Additional file 1). In the interest of expediting dissemination of this material, the familiar formatting has been eliminated; this Letter serves as a summary of the key elements of the full protocol.

**Supplementary Information:**

The online version contains supplementary material available at 10.1186/s13063-020-04840-y.

## Supplementary Information

**Additional file 1.** Full Study Protocol.

## Data Availability

The final trial data will be available from the author on reasonable request (Email address of corresponding author is deeptikailath@gmail.com).

