# Supplementary material for: Atorvastatin and Aspirin as Adjuvant Therapy in Patients with SARS-CoV-2 Infection: A structured summary of a study protocol for a randomised controlled trial
Source: Trials. 2020 Oct 30;21:902. doi: 10.1186/s13063-020-04840-y (PMC7598224; doi:10.1186/s13063-020-04840-y)

# A Randomised Control Trial of Statin and Aspirin as Adjuvant Therapy in Patients with SARS-CoV-2 Infection (*RESIST Trial*)

***Research Protocol***
***(COVID-19)***

**JUNE 2020**

**AIIMS, NEW DELHI** **DR SIDDHARTHAN DEEPTI**

# Introduction

A series of cases with acute atypical respiratory illnesses was reported from Wuhan, China in December 2019. This disease has rapidly spread from Wuhan to other areas. The causative agent was found to be a novel coronavirus named as the severe acute respiratory syndrome coronavirus-2 (SARS-CoV-2, 2019-nCoV). The disease was called Coronavirus disease 19 (COVID-19) and a pandemic was declared by the World Health Organization (WHO). COVID-19 is rapidly spreading and as of June 14th, 2020, more than 7.8 million cases worldwide have been reported according to the Center for Systems Science and Engineering (CSSE) at John Hopkins University (1).

SARS-CoV-2 virus primarily affects the respiratory system leading to extremely heterogenous clinical profiles ranging from minimal symptoms to significant hypoxia with acute respiratory distress syndrome (ARDS). The clinical manifestations and the severity of illnesses following COVID-19 infections are the result of immune dysregulations. Immunological studies in patients with severe COVID-19 infection have shown increased plasma concentrations of proinflammatory cytokines, including interleukin (IL)-6, IL-10, granulocyte-colony stimulating factor (G-CSF), monocyte chemoattractant protein 1 (MCP1), macrophage inflammatory protein (MIP)1$\alpha$, and tumor necrosis factor (TNF)-$\alpha$ (2). The levels of IL-6 increased with the severity of the illness. There was increased activation and subsequent exhaustion of CD4+ and CD8+ T cells which has been proposed as a potential contributor to the progression of the disease (2).

The interval between the onset of symptoms and the development of ARDS has been observed to be as short as 9 days, suggesting a rapid progression of respiratory symptoms in some cases. The disease has a combined case-fatality rate of 2.3% with >430,000 deaths reported globally (3,4). A growing number of patients with the severe form of the disease are continuing to succumb worldwide. Current treatment is largely supportive with no targeted therapy available.

There is an imperative need for the development of antiviral and immunomodulatory drugs that can modify the course of the disease. Several drugs including lopinavir-ritonavir, remdesivir, hydroxychloroquine, azithromycin and ivermectin are being evaluate in clinical trials with no promising result so far. This necessitates consideration of the option of adjuvant drug therapy with immunomodulatory and antiviral properties. In this respect, an ideal adjuvant drug should be a low-cost generic medication, easily accessible in pandemics events with an established safety profile. This type of drug should aim at the host's immune response to alleviate the effects of immune dysregulation. Such anti-inflammatory and immunomodulatory agents may effectively complement the usual care in COVID-19 infection. It is with this premise that we intend to evaluate two commonly used cardioactive drugs with anti-inflammatory properties, statins and aspirin, as adjuvant therapy in COVID-19 infection.

# Review of literature

## Statins

Beyond their hypolipidemic benefits, statins exert multiple cholesterol-independent pharmacological effects (5). These pleiotropic actions of statins include anti-inflammatory, antioxidant, anti-proliferative and immunomodulatory effects, normalization of sympathetic outflow, and prevention of platelet aggregation. Pleiotropy of statins have been proposed to account for the fact that the overall cardiovascular benefits of statins are larger than what one would expect based solely on the reduction in lipid levels.

The underlying mechanism for anti-inflammatory actions of statins involves reduction in farnesyl and geranylgeranyl residues as a by-product of inhibition of HMG-CoA reductase. These residues are responsible for the correct attachment of different small GTPases to the cell membrane. The blockade of this attachment, GTPase isoprenylation, affects the immune response at multiple levels including T-cell signalling, antigen presentation, immune cell migration and cytokine production.

In-vitro studies have shown inhibition of cytokine production including IL-6, IL-8, and GM-CSF by statins, independent of the cholesterol synthesis pathway (6). IL-6 is a pleiotropic cytokine which serves as a critical inflammatory mediator in inflammatory lung diseases. IL-6 plays a role in the production of C-reactive protein which is involved in organ dysfunction and death in critically ill patients.

In mouse models of endotoxin-induced acute lung injury (ALI) to study oxidative stress, statins have been found to significantly reduce levels of redox markers (superoxide dismutase and catalase), the extent of lipid peroxidation (malondialdehyde and hydroperoxides) and myeloperoxidase (7). The antioxidant effects of statins also relate to their ability to inhibit the production of isoprenoid compounds via the mevalonate pathway and consequential downregulation of redox-sensitive proinflammatory transcriptional factors such as NF-ϰB.

Based on these pleiotropic effects, statins have been evaluated as an immunomodulatory agent in bacterial and viral pneumonias. Observational studies have shown that prior therapy with statins may be associated with a reduced rate of severe sepsis and ICU admission in patients with acute bacterial infections (8,9). A large population-based, retrospective study showed that patients on statins had a significantly reduced risk of fatal pneumonia and slightly but not significantly reduced risks of uncomplicated pneumonia and pneumonia hospitalization with survival. A randomised comparison of pravastatin with a placebo (n=44) for reducing ventilator-associated pneumonia (VAP) in patients on mechanical ventilation and intensive care unit stay of >48 hours showed an indication for increased probability of being free from VAP during the 30-day treatment period in the pravastatin group (10). On the contrary, another randomised study failed to show a benefit on 28-day survival of addition of simvastatin In adults with suspected VAP (11).

The observations are more promising in viral pneumonias. A summary of studies on use of statins in in-vitro and murine models of viral pneumonias is provided in Table 1. A recently completed randomised controlled trial (ClinicalTrials.gov number, NCT02056340) showed a significant improvement of symptoms in statin-naïve patients hospitalized for seasonal influenza receiving atorvastatin compared with placebo.

Specific to coronaviruses, statins have some actions that might be especially beneficial. SARS-CoV-1 interacts with Toll-like receptors on the host cell membrane and significantly induces the expression of the MYD88 gene (12). Downstream effects of this include activation of the NF-kB pathway and severe inflammation, a hallmark of COVID-19 infection. Statins have been shown to stabilize MYD88 levels after a proinflammatory trigger in experimental models, thereby attenuating the inflammatory response (13).

A second theoretical anti-coronavirus action of statins involves interference with ACE2 signalling. SARS-CoV-2 utilizes ACE2 for initial entry and then down-regulates ACE2

expression. This action possibly facilitates the initial influx of innate immunity cells, causing an unopposed angiotensin II accumulation and consequent organ injury (14). Statins are known to experimentally up-regulate ACE2 via epigenetic modifications (15). Since an increase in ACE2 might prove beneficial for COVID-19 patients, there is biological plausibility to investigate statins in decreasing the severity of COVID-19 infection.

Table 1: Table showing existing literature on statins as anti-inflammatory and anti-viral agent

| Authors | Agent | Model | Target | Observations |
|---|---|---|---|---|
| In Vitro Studies | | | | |
| Haidari et al(16) | Atorvastatin Rosuvastatin | Madin-Darby canine kidney (MDCK) cells | H3N2 and H1N1 strains of influenza | Inhibition of influenza virus proliferation by downregulating the Rho/Rho kinase pathway |
| Mehrbod et al(17) | Atorvastatin, Simvastatin Pravastatin | Crandell feline kidney (CrFK) cells | H1N1 strain | Significant reduction in the expression of proinflammatory cytokine proteins, such as TNF- and IL-6 in atorvastatin-, simvastatin-, and pravastatin-treated H1N1 infected cells compared to the virus infected cells alone |
| Lee et al(18) | Simvastatin | Primary normal human bronchial epithelial cells (NHBE)<br>Human type II pneumocyte cell line A549 | Polyinosinic–polycytidylic acid (poly I:C)-induced airway inflammation | Attenuation of viral dsRNA-induced AKT phosphorylation, STAT3 activation, and the subsequent production of RANTES |

शरीरमाद्यं खलु धर्मसाधनम्

| | | | | |
|---|---|---|---|---|
| Mehrbod et al(19) | Atorvastatin | Madin-Darby canine kidney (MDCK) cells | H1N1 strains of influenza | Reduction in viral titres |
| Animal studies | | | | |
| Gower et al(20) | Lovastatin | Murine model | Respiratory syncytial virus | Modification of RhoA membrane localization thereby affecting virus replication |
| Haidari et al(21) | Atorvastatin | Murine model | H3N2 and H1N1 | Reduction in lung virus titers and mortality rates |
| Liu et al(22) | 50 *Mg* Statin/200 *Mg* Caffeine Mixture | Murine model | H5N1, H3N2, and H1N1 influenza virus infections | Inhibition of virus replication and improvement in lung damage |
| Kumaki et al(23) | Simvastatin, Lovastatin, Mevastatin, Pitavastatin, Atorvastatin Rosuvastatin | Murine model | H5N1, H3N2, and H1N1 influenza virus infections | No significant improvement in survival |

| | | | | |
|---|---|---|---|---|
| Belser et al(24) | Simvastatin | Murine model | H5N1 or pH1N1 | No prominent antiviral activity of simvastatin alone or in combination therapy<br>Reduced hypercytokinemia following H5N1 but not pH1N1 infections |

## Aspirin

Aspirin or acetylsalicylic acid (ASA) is one of the most commonly used anti-platelet drug for the prevention of myocardial infarction, stroke, cardiac stent thrombosis, and bypass graft protection. It irreversibly inhibits platelet cyclooxygenase 1 and 2 (COX-1/-2) enzymes. This results in decreased synthesis of thromboxane A2 leading to an inhibitory effect on platelet aggregation (24, 25). In addition to its anti-platelet effects, aspirin has also been used as an anti-inflammatory drug in several immune-mediated diseases (e.g., acute rheumatic fever) (26). Few in-vitro studies have also proposed an anti-viral property of aspirin against several viruses like- hepatitis C (27,28), varicella zoster (29), cytomegalovirus (30), influenza A virus (31). Though the exact mechanism behind its anti-inflammatory and anti-viral action is still unknown, several hypotheses have been proposed (32) – first, aspirin causes uncoupling of oxidative phosphorylation in hepatic mitochondria (33); second, it induces NO radicals responsible for a decrease in inflammation (34); third, it modulates signalling through transcription factor NF-kB which play central role in many biological processes including systemic inflammation. In addition, aspirin modulates claudin-1 receptor in host cell membrane inhibiting hepatitis C virus entry (35). One in-vivo experiment with Influenza A virus has also suggested aspirin's anti-viral action through its influence on the NF-kB pathway (36).

Aspirin is one of the potential drugs that are being tested in multiple ongoing trials including one large RCT (Clinicaltrials.gov, NCT0433340), as an adjuvant anti-inflammatory, anti-viral, and anti-platelet therapy against SARS-CoV-2 virus infection. Recent electron microscopic and immunohistochemical evidence has suggested an elevated number of megakaryocytes and platelet-rich thrombi in alveolar capillaries from lung tissue of deceased patients with severe COVID-19 infection (37). Aspirin may inhibit the formation of platelet rich thrombi leading to an improvement in pulmonary regional ventilation-perfusion mismatch. Subsequent decrease in the alveolar capillary membrane thickness may also improve oxygen diffusion capacity and

oxygen saturation in patients with ARDS due to severe COVID-19 infection (38, 39). Till date there is only one case control study (39) that evaluated the benefit of adding antiplatelet agents in addition to the prophylactic anticoagulants in the management protocol of severe COVID-19 infection. In that study, tirofiban infusion followed by aspirin and clopidogrel combination for 30 days resulted in significant improvement in A-a O2 gradient in COVID-19 patients with severe hypoxemia. Moreover, there was a significant improvement in ARDS resulting in weaning from CPAP therapy in patients who received antiplatelet agents. However, the current evidences on aspirin is very scarce to either support or defy its regular use in the management of COVID-19 infected patients.

ALL INDIA INSTITUTE OF MEDICAL SCIENCES

# Method and Materials

**Research Questions:**

1) Is there any mortality and morbidity benefit of adding statin to usual care in SARS-CoV-2 infected patients?
2) Is there any mortality and morbidity benefit of adding aspirin to usual care in SARS-CoV-2 infected patients?
3) Does addition of both aspirin and statin to usual care in patients of COVID-19 result in any mortality and morbidity benefit?
4) What are the safety concerns, if any, of statin and aspirin therapy in SARS-CoV-2 infected patients?

**Study Design:** Single-centre, prospective, four-arm parallel design, open-label randomized control superiority trial.

**Sample Size:** As there is no existing study that have evaluated the role of statin and atorvastatin in patients infected with SARS-CoV-2, formal sample size calculation has not been done. Patients satisfying the inclusion and inclusion criteria will be recruited in the study during six months of study period. Once first 200 patients are included in each arm (i.e., total 800 patients), final sample size calculation will be done on the basis of the interim analysis of the collected data.

**Place of Study:**

The study will be conducted at the National Cancer Institute (NCI), Jhajjar, Haryana, which is a part of the All India Institute of Medical Sciences (AIIMS), New Delhi, and has been converted into a dedicated COVID-19 management centre since the outbreak of the pandemic.

**Study Population:**

All SARS-CoV-19 infected patients requiring admission in the study centre will be screened for the trial.

<u>Inclusion criteria:</u>

- Age ≥ 40 years, < 75 years
- RT-PCR positive for SARS-CoV-19 infection
- Symptoms (WHO clinical improvement ordinal score 3 to 5) requiring hospital admission
- Consenting to participate for the trial

<u>Exclusion criteria:</u>

- Critical illness with WHO clinical improvement ordinal score > 5
- Documented significant liver disease / dysfunction (AST/ALT > 240)
- Myopathy and Rhabdomyolysis (CPK > 5x normal)
- Allergy or intolerance to statins
- Allergy or intolerance to aspirin
- Patients taking the following medications: cyclosporine, HIV protease inhibitors, hepatitis C protease inhibitor, telaprevir, fibric acid derivatives (gemfibrozil), niacin, azole antifungals (itraconazole, ketoconazole) clarithromycin and colchicine
- Prior statin use (within 30 days)
- Prior aspirin use (within 30 days)
- History of active GI bleeding in past three months
- Coagulopathy
- Thrombocytopenia (Platelet count < 100000/ dl)
- Pregnancy, active breast-feeding

- Patient unable to take oral or nasogastric medications

**Patient recruitment and baseline investigations:**

All confirmed (RT-PCR) and mild-moderately symptomatic cases of SARS-CoV-19 infection requiring admission in the National Cancer Institute (NCI), Jhajjar, Haryana will be screened for recruitment. The patients will be managed according to the institute treatment protocol (**Appendix – III**). Demographic information, including age, gender, residential address, BMI will be recorded in a structured proforma (**Table 2 and Appendix-I**). All patients will be clinically evaluated for comorbidities (e.g., diabetes mellitus, hypertension, coronary artery disease, heart failure, ischemic stroke, chronic kidney disease, chronic liver disease, etc.), chronic medication history, COVID-19 related symptoms and signs (e.g., fever, cough, sore throat, dyspnea, body ache, etc.). Routine investigations like chest x-ray, 12-lead electrocardiography (ECG), complete blood count, liver function test, renal function test, fasting blood sugar- will be documented at baseline.

**Randomization and intervention:**

The study will use a four-arm parallel group design. A computer-generated permutated block randomization with mixed block size will be used to randomize the participants in a 1:1:1:1 ratio to the group A (Atorvastatin with conventional therapy), group B (Aspirin with conventional therapy), group C (Aspirin + Atorvastatin with conventional therapy), and group D (Control; only conventional therapy). Atorvastatin will be prescribed as 40mg oral tablets once daily for 10 days or until discharge whichever is earlier. Aspirin dose will be 75mg once daily for 10 days or until discharge whichever is earlier.

**Follow up:**

All study participants will be prospectively followed up for ten days or until hospital discharge whichever is longer for outcomes. Patients with early discharge (due to clinical improvement and patient's preference for home isolation) will be followed up by alternate day telephonic contact till $10^{th}$ day of drug regimen. Serum CPK, LFT, Trop -I, serum inflammatory biomarkers – i.e., CRP, and IL-6 will be repeated on $5_{th}$ day of study enrolment or $7_{th}$ day after

symptom onset, whichever is later. Decision regarding other medications and investigation will be based on institute management protocol and treating physician's clinical judgement.

**Table 2:** Information to be recorded during the study

| | | |
|---|---|---|
| Demographic factors | • Age<br>• Gender<br>• Address<br>• Mobile number<br>• BMI | |
| Comorbidities | • Hypertension<br>• Diabetes<br>• Dyslipidemia<br>• Coronary artery disease<br>• Left ventricular dysfunction | • Ischemic stroke<br>• Chronic kidney disease |
| Covid-19 related symptoms | • Fever<br>• Cough<br>• Sore-throat | • Body ache<br>• GI symptoms<br>• Others |
| Investigations | • CBC<br>• LFT<br>• RFT<br>• CXR | • CRP<br>• IL-6<br>• Trop-I |

| | | |
|---|---|---|
| | • Fasting blood sugar | |
| Drugs | • Hydroxychloroquine<br>• Azithromycin<br>• Other antibiotics<br>• Antiviral<br>• Paracetamol<br>• Vitamin C | • Pantoprazole<br>• Plasma therapy<br>• Tocilizumab<br>• Other medications |

**Outcome Variables:**

Primary

- Clinical deterioration characterised by progression to WHO clinical improvement ordinal score ≥ 6 (i.e., endotracheal intubation, non-invasive mechanical ventilation, pressor agents, RRT, ECMO, and mortality).

Secondary

- Change in serum inflammatory markers (CRP, and IL-6), Trop I, CPK from time zero to 5th day of study enrolment or 7th day after symptom onset, whichever is later.

Other outcomes

- Progression to ARDS
- Progression to shock
- ICU admission
- Length of hospital stay
- Length of ICU stay
- In-hospital mortality

Safety concerns

- Myalgia – severe muscle pain or aches (CPK < ULN)
- Myopathy - unexplained muscle pain or weakness accompanied by CPK >10x ULN
- Rhabdomyolysis - severe myopathy with CPK >40x ULN and myoglobinuria ± acute renal failure
- Hepatotoxicity (ALT/AST > 3x ULN; hyperbilirubinemia)
- Minor Bleeding (BARC bleeding type 1 and 2 - Bleeding that is not actionable and does not cause the patient to seek treatment, bleeding requiring a healthcare assessment or less invasive treatment - such as heavy menstrual bleeding, ecchymosis, or epistaxis etc.)
- Major Bleeding (BARC bleeding type ≥ 3 - significant blood loss requiring blood transfusion, bleeding into a critical closed space [eg, intracranial bleeding, compartment syndrome], bleeding requiring an intervention for management [eg, surgery, interventional radiology procedures, endoscopic treatments], fatal bleeding)
- Other

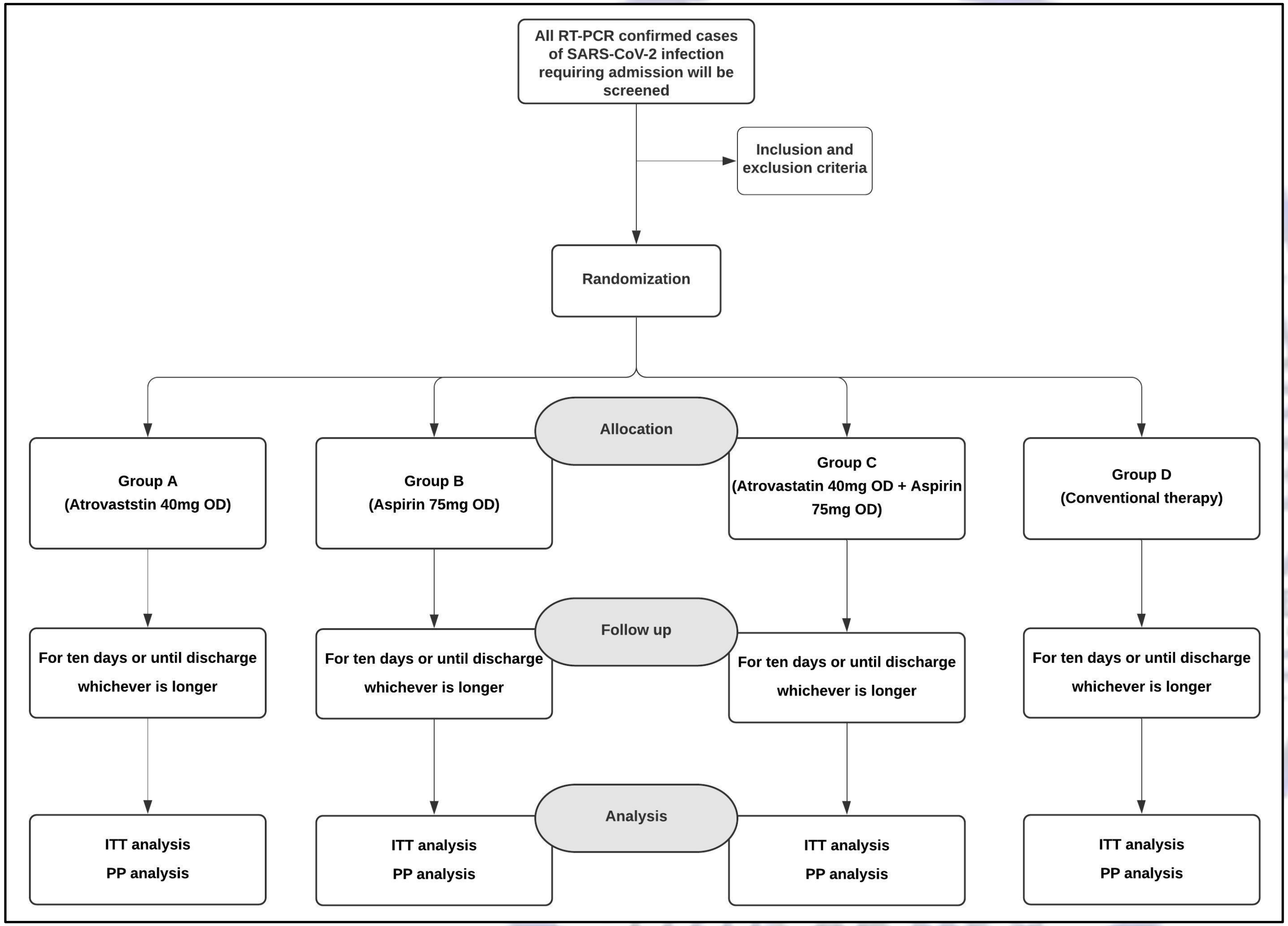


**Figure 1:** Flow diagram summarizing the study method

शरीरमाद्यं खलु धर्मसाधनम्

**Statistical Analysis:**

The quantitative variables will be summarized through descriptive statistics, i.e., mean (±SD) or interquartile range, and the categorical variables will be summarized through frequency (%). Both Intention-to-treat (ITT) and Per Protocol (PP) analysis will be carried out for primary and secondary outcomes. The primary outcome will be compared between the groups using proportions test. Serum inflammatory markers will be test for normality assumption using Shapiro-Wilks test. Variables that follow normal distribution will be compared between the groups over a period of time using generalized estimating equation and those variables that do not follow normal will be analyzed using Wilcoxon rank sum test and Wilcoxon signed rank test. Other outcomes (time-to-event) will be compared using Kaplan-Meier curve and log-rank test. The Cox proportional hazards model will be used to calculate hazards ratio and 95% Confidence Interval. Safety outcomes will be compared between the groups using Chi-square or Fisher's exact test. Two-sided P value <0.05 will be considered significant. The data will be assessed using STATA statistical software.

ALL INDIA INSTITUTE OF MEDICAL SCIENCES
शरीरमाद्यं खलु धर्मसाधनम्

# References


1. JHUoMCr center Journal. 2020 https://coronavirus.jhu.edu/map.html
2. Zheng M., Gao Y., Wang G., Song G., Liu S., Sun D., Xu Y., Tian Z. Functional exhaustion of antiviral lymphocytes in COVID-19 patients. Cell Mol Immunol. 2020
3. Wu Z., McGoogan J.M. Characteristics of and important lessons from the Coronavirus disease 2019 (COVID-19) outbreak in China: summary of a report of 72314 cases from the Chinese Center for Disease Control and Prevention. JAMA. 2020
4. Onder G., Rezza G., Brusaferro S. Case-fatality rate and characteristics of patients dying in relation to COVID-19 in Italy. JAMA. 2020
5. Liao JK. Clinical implications for statin pleiotropy. Current opinion in lipidology. 2005 Dec 1;16(6):624-9.
6. Iwata A, Shirai R, Ishii H et al. Inhibitory effect of statins on inflammatory cytokine production from human bronchial epithelial cells. Clinical & Experimental Immunology. 2012 May;168(2):234-40.
7. Melo AC, Valença SS, Gitirana LB, et al. Redox markers and inflammation are differentially affected by atorvastatin, pravastatin or simvastatin administered before endotoxin-induced acute lung injury. International immunopharmacology. 2013 Sep 1;17(1):57-64.
8. Almog Y, Shefer A, Novack V, et al. Prior statin therapy is associated with a decreased rate of severe sepsis. Circulation. 2004;110:880–5.
9. Dobesh PP, Klepser DG, McGuire TR, Morgan CW, Olsen KM. Reduction in mortality associated with statin therapy in patients with severe sepsis. Pharmacotherapy. 2009;29:621–30.

10. Makris D, Manoulakas E, Komnos A, et al. Effect of pravastatin on the frequency of ventilator-associated pneumonia and on intensive care unit mortality: open-label, randomized study. Critical care medicine. 2011;39(11):2440-6.
11. Papazian L, Roch A, Charles PE, et al Effect of statin therapy on mortality in patients with ventilator-associated pneumonia: a randomized clinical trial. Jama. 2013;310(16):1692-700.
12. Totura AL, Whitmore A, Agnihothram S, et al. Toll-like receptor 3 signaling via TRIF contributes to a protective innate immune response to severe acute respiratory syndrome coronavirus infection. MBio. 2015;6(3):e00638-15.
13. Yuan X, Deng Y, Guo X, et al. Atorvastatin attenuates myocardial remodeling induced by chronic intermittent hypoxia in rats: partly involvement of TLR-4/MYD88 pathway. Biochemical and biophysical research communications. 2014;446(1):292-7.
14. Madjid M, Safavi-Naeini P, Solomon SD, et al. Potential effects of coronaviruses on the cardiovascular system: a review. JAMA cardiology. 2020 Mar 27.
15. Fedson DS, Opal SM, Rordam OM. Hiding in plain sight: an approach to treating patients with severe COVID-19 infection. Mbio. 2020 Apr 28;11(2).
16. M. Haidari, A. Muzammil, S. W. Casscells, et al. "Statins block influenza infection by down-regulating Rho/Rho kinase pathway," Circulation 2007;116;116-117
17. Mehrbod P, El Zowalaty M, Omar AR, Hair-Bejo M, Ideris A. Statins reduce the expression of proinflammatory cytokines in influenza A virus infected CrFK cells. Acta Virol. 2012;56(4):353-5.
18. Lee CS, Yi EH, Lee JK, et al. Simvastatin suppresses RANTES-mediated neutrophilia in polyinosinic–polycytidylic acid-induced pneumonia. European Respiratory Journal. 2013;41(5):1147-56.

19. Mehrbod P, Ideris A, Omar AR, Hair-Bejo M. Evaluation of antiviral effect of atorvastatin on H1N1 infection in MDCK cells. Afr J Mic Res. 2012 Jul 19;6:5715-9.
20. Gower TL, Graham BS. Antiviral activity of lovastatin against respiratory syncytial virus in vivo and in vitro. Antimicrobial agents and chemotherapy. 2001 Apr 1;45(4):1231-7.
21. Liu Z, Guo Z, Wang G, et al. Evaluation of the efficacy and safety of a statin/caffeine combination against H5N1, H3N2 and H1N1 virus infection in BALB/c mice. European journal of pharmaceutical sciences. 2009;38(3):215-23.
22. Kumaki Y, Morrey JD, Barnard DL. Effect of statin treatments on highly pathogenic avian influenza H5N1, seasonal and H1N1pdm09 virus infections in BALB/c mice. Future virology. 2012;7(8):801-18.
23. Belser JA, Szretter KJ, Katz JM, et al. Simvastatin and oseltamivir combination therapy does not improve the effectiveness of oseltamivir alone following highly pathogenic avian H5N1 influenza virus infection in mice. Virology. 2013;439(1):42-6.
24. Baigent C, Blackwell L, Collins R, Emberson J, Godwin J, Peto R, et al. Aspirin in the primary and secondary prevention of vascular disease: collaborative meta-analysis of individual participant data from randomised trials. Lancet. 2009;373(9678):1849-60.
25. Ishida K, Messé SR. Antiplatelet strategies for secondary prevention of stroke and TIA. Curr Atheroscler Rep. 2014;16(11):449.
26. Morris T, Stables M, Hobbs A, de Souza P, Colville-Nash P, Warner T, et al. Effects of low-dose aspirin on acute inflammatory responses in humans. J Immunol. 2009;183(3):2089-96.
27. Sánchez-García A, Ríos-Ibarra CP, Rincón-Sánchez AR, Ortiz-López R, Garza-Juárez A, Morlett-Chávez J, et al. Use of proteomic analysis tools to identify HCV-proteins down-regulated by acetylsalicylic acid. Ann Hepatol. 2013;12(5):725-32.

ALL INDIA INSTITUTE OF MEDICAL SCIENCES
शरीरमाद्यं खलु धर्मसाधनम्

28. Trujillo-Murillo K, Rincón-Sánchez AR, Martínez-Rodríguez H, Bosques-Padilla F, Ramos-Jiménez J, Barrera-Saldaña HA, et al. Acetylsalicylic acid inhibits hepatitis C virus RNA and protein expression through cyclooxygenase 2 signaling pathways. Hepatology. 2008;47(5):1462-72.
29. Primache V, Binda S, De Benedittis G, Barbi M. In vitro activity of acetylsalicylic acid on replication of varicella-zoster virus. New Microbiol. 1998;21(4):397-401.
30. Speir E, Yu ZX, Ferrans VJ, Huang ES, Epstein SE. Aspirin attenuates cytomegalovirus infectivity and gene expression mediated by cyclooxygenase-2 in coronary artery smooth muscle cells. Circ Res. 1998;83(2):210-6.
31. Huang RT, Dietsch E. Anti-influenza viral activity of aspirin in cell culture. N Engl J Med. 1988;319(12):797.
32. Glatthaar-Saalmüller B, Mair KH, Saalmüller A. Antiviral activity of aspirin against RNA viruses of the respiratory tract-an in vitro study. Influenza Other Respir Viruses. 2017;11(1):85-92.
33. Somasundaram S, Sigthorsson G, Simpson RJ, Watts J, Jacob M, Tavares IA, et al. Uncoupling of intestinal mitochondrial oxidative phosphorylation and inhibition of cyclooxygenase are required for the development of NSAID-enteropathy in the rat. Aliment Pharmacol Ther. 2000;14(5):639-50.
34. Paul-Clark MJ, Van Cao T, Moradi-Bidhendi N, Cooper D, Gilroy DW. 15-epi-lipoxin A4-mediated induction of nitric oxide explains how aspirin inhibits acute inflammation. J Exp Med. 2004;200(1):69-78.
35. Yin P, Zhang L. Aspirin inhibits hepatitis C virus entry by downregulating claudin-1. J Viral Hepat. 2016;23(1):62-4.

36. Mazur I, Wurzer WJ, Ehrhardt C, Pleschka S, Puthavathana P, Silberzahn T, et al. Acetylsalicylic acid (ASA) blocks influenza virus propagation via its NF-kappaB-inhibiting activity. Cell Microbiol. 2007;9(7):1683-94.
37. Carsana L, Sonzogni A, Nasr A, Rossi RS, Pellegrinelli A, Zerbi P, et al. Pulmonary post-mortem findings in a series of COVID-19 cases from northern Italy: a two-centre descriptive study. The Lancet Infectious Diseases. 2020.
38. Petersson J, Glenny RW. Gas exchange and ventilation-perfusion relationships in the lung. Eur Respir J. 2014;44(4):1023-41.
39. Viecca M, Radovanovic D, Forleo GB, Santus P. Enhanced platelet inhibition treatment improves hypoxemia in patients with severe Covid-19 and hypercoagulability. A case control, proof of concept study. Pharmacol Res. 2020;158:104950.

ALL INDIA INSTITUTE OF MEDICAL SCIENCES
शरीरमाद्यं खलु धर्मसाधनम्

## Appendix I (Study Proforma):

| | |
|---|---|
| Name- | |
| Age – | Height - |
| Sex – | Weight - |
| UHID no.- | BMI - |
| Address – | Date of randomization- |
| Mobile No. – | Date of symptom onset - |
| Date of admission – | |
| Date of discharge – | |
| Date of positive RT-PCR - | |

| Comorbidities | Yes/No |
|---|---|
| Hypertension | |
| Diabetes | |
| Coronary artery disease | |
| Dyslipidemia | |
| Left ventricular dysfunction | |
| Chronic kidney disease | |
| Chronic liver disease | |
| Other comorbidities | |

| Covid-19 related symptoms | Yes/No |
|---|---|
| Fever | |
| Cough | |
| Dyspnea | |
| Desaturation | |
| Sore throat | |
| Body ache | |
| GI symptoms | |
| Others | |

| Drugs | Yes/No |
|---|---|
| Hydroxychloroquine | |
| Azithromycin | |
| Other antibiotics | If yes - specify |
| Anti-viral | If yes - specify |
| Pantoprazole | |
| Paracetamol | |
| Vitamin C | |
| Anticoagulants | |

शरीरमाद्यं खलु धर्मसाधनम्
ALL INDIA INSTITUTE OF MEDICAL SCIENCES

| | |
|---|---|
| Plasma therapy | |
| Tocilizumab | |
| Steroid | |
| Others | |

| **Investigations** | **Day 0 (pre-statin therapy)** | **Date** | **Date** |
|---|---|---|---|
| HB | | | |
| TLC | | | |
| DLC | | | |
| Platelet | | | |
| Urea | | | |
| Creatinine | | | |
| Total bilirubin | | | |
| Direct bilirubin | | | |
| SGOT | | | |
| SGPT | | | |
| ALP | | | |
| PT | | | |
| D-dimer | | | |
| Ferritin | | | |
| Fasting sugar | | | |

| **Primary outcome** | **WHO ordinal score** |
|---|---|
| Baseline | |
| Final / Maximum | |
| WHO ordinal scale ≥ 6 (Yes/no) | |

| Secondary & safety outcomes | | |
|---|---|---|
| **Serum biomarkers** | **Day 0 (before statin therapy)** | **Day 5 of enrolment (/ Day 7 of symptom)** |
| CRP | | |
| IL-6 | | |
| Trop I | | |
| SGOT | | |
| SGPT | | |
| ALP | | |
| CPK | | |

| Other adverse outcomes | Yes/No | If yes specify |
|---|---|---|
| ARDS | | |
| Shock | | |
| Mechanical ventilation | | |
| ICU admission | | |
| Length of hospital stay | | ? Number of days |
| Length of ICU stay | | ? Number of days |
| In-hospital mortality | | |

| Safety concerns | Yes/No | If yes- date | Other specify |
|---|---|---|---|
| Myalgia | | | |
| Myopathy | | | |
| Rhabdomyolysis | | | |

| | | | |
|---|---|---|---|
| Hepatotoxicity | | | |
| Minor Bleeding | | | |
| Major Bleeding | | | |
| Others | | | |

# Appendix II

## Patient Information Sheet

**Title of the Study/Project**

A Randomised Control Trial of Statin and Aspirin as Adjuvant Therapy in Patients with SARS-CoV-2 Infection (RESIST Trial)

**Background of the study**

As you must be aware, you have been diagnosed with COVID-19, an ongoing worldwide pandemic. COVID-19 stands for "Coronavirus disease 2019." It is caused by a virus called SARS-CoV-2. The virus first appeared in late 2019 and quickly spread around the world. People with COVID-19 can have fever, cough, troubled breathing, and other symptoms. Most people who get COVID-19 will not get severely ill. But some people do so. Problems with breathing happen when the infection affects the lungs and causes pneumonia. Severe form of such lung disease may occur and is called Acute respiratory distress syndrome (ARDS) which is the major complication. It manifests shortly after the onset of pneumonia or lung infection in 7-8% of patients. Many other complications can occur including clotting in blood vessels, heart injury, kidney injury, and widespread inflammation in body. Till date there is no known specific treatment for COVID-19. Atorvastatin (Statin) and aspirin are two medicines that are widely used in prevention of heart and brain attacks. These two drugs are being studied in COVID-19 patients all over the world due to their encouraging anti-viral and anti-inflammatory actions that may prevent lung complications and serious outcomes including death. There is currently very little data available on their efficacy and safety in COVID-19 patients.

**Aims and methods of the research**

This research is being done to assess the utility and safety of atorvastatin (Statin) and aspirin in COVID-19 patients. This study will help to learn whether these widely available medicines are useful or not in management and preventing complications of COVID-19 patients.

If you consent, you will be enrolled as a subject in the study during your hospital admission. The doctor will take brief history followed by focused physical examination. Then you will be allotted to one of the four groups i.e., Group A (Will receive Atorvastatin tablet), Group B (Will receive Aspirin tablet), Group C (Will receive both Aspirin tablet and Atorvastatin tablet), Group D (Will receive only conventional therapy). The allotment will be done randomly with the help of a computer – not by your or investigator's choice. The decision to

prescribe other conventional medicines, as well as all other aspects of your treatment will be decided by your treating doctor as per AIIMS protocol and will not be affected by your taking part in this study. Approx. 10 ml of blood (about 2 teaspoon) will be drawn on the day of enrolment for routine blood tests like complete blood count, kidney function test, liver function test, lipid profile etc. which will be helpful for both the disease management and the research. Aspirin and atorvastatin will be given for ten days or till your discharge whichever is later. You will be followed up for ten days or till discharge, whichever is later, during which repeat blood tests will be done on $5^{th}$ day (for trial) and as required for your treatment.

**Expected duration of the subject participation**

As mentioned above the duration of the study would be ten days or till your discharge whichever is earlier.

**Maintenance of confidentiality of records**

In order to preserve your confidentiality only an anonymous subject number will be associated with the information you provide. Your name will not appear on any publication or be released to anyone without your written consent. Your anonymised records will be kept secured and confidential.

**Cost of research related drugs and investigations**

The drugs being tested in the trial will be provided free of cost. Also, the blood tests to be done as part of this research project will be performed free of cost.

**Any risk to the subject associated with the study**

Both atorvastatin and aspirin are time-tested and safe drugs. Atorvastatin has been used to lower bad cholesterol (LDL), prevent heart attack, brain attack; and aspirin to prevent heart attack, stroke and protect stents and bypass grafts in enumerable patients without any major safety concerns. Atorvastatin can very rarely cause muscle pain, muscle injury, muscle breakdown (0.005-0.01%), and liver dysfunction (0.5-1%). However, most of these side-effects occur with prolonged use. The probability of these side-effects with 10-day use is very low. Aspirin may also result in allergies (2.5%) and major bleeding (0.05%). However, the probability at low dose and with ten days use is again extremely low. During the study we will closely monitor you for these side-effects and will immediately stop the drugs when necessary. Appropriate treatment will be provided at our hospital in the extremely unlikely situation that

any of these side-effects occur. The investigations needed for the study are benign and do not have any potential to cause harm to the patient.

**Benefits**

Your participation will help us to assess the role of aspirin and atorvastatin in preventing complications like Acute respiratory distress syndrome (ARDS), shock (severely low blood pressure), and other COVID-19 related major complications. This information will help in creating an effective and safe management strategy for COVID-19 patients in future.

**Provision of free treatment for research related injury and compensation of subjects for disability or death resulting from such injury**

Appropriate treatment free of cost will be provided at our hospital, AIIMS, New Delhi in the extremely unlikely situation that any of the above-mentioned side-effects occur. However, this being an investigator led academic study no compensation will be provided in the very unlikely treatment related disability or death arising out of the study.

Your participation in the study is completely voluntary. Choosing not to participate will not affect treatment services you may be eligible for now or in the future. You can also leave the study at any time without giving any reason if you wish to. This would not lead to any penalty or loss of benefit to you as a patient of this hospital. You can ask questions about this project at any time. You may contact the investigators given below, if you have any questions or grievances about this research study.

Please feel free to ask about anything you do not understand. Please read and review this research and patient information form carefully before you agree to participate. You may take as much time as you need to think over it.

ALL INDIA INSTITUTE OF MEDICAL SCIENCES
शरीरमाद्यं खलु धर्मसाधनम्

## <u>Contact addresses</u>

| **Dr Nirmal Ghati** | **Dr Deepti Siddharthan** |
|---|---|
| Senior Resident<br>Department of Cardiology, AIIMS, New Delhi<br>Phone: 9013860076<br>E mail: nirmal.cmc@gmail.com | Assistant Professor<br>Department of Cardiology, AIIMS, New Delhi<br>Phone: 9968774019<br>E mail: deeptikailath@gmail.com |
| **Questions regarding your rights as a volunteer may be addressed to the committee that reviewed the ethical aspects of this study at Phone number – 011-2654579.** | |

# INFORMED CONSENT FORM

Protocol/Study No.: ______________________________

Patient Id No. for this trial: _______________________

**Project Title: "A Randomised Control Trial of Statin and Aspirin as Adjuvant Therapy in Patients with SARS-CoV-2 Infection (RESIST Trial)"**

**Principle Investigator: Dr Deepti Siddharthan Phone: 9968774019**

**Co-Investigator: Dr Nirmal Ghati Phone: 9013860076**

The content of information sheet dated ________ (version) ___________ that was provided have been read carefully by me/explained to me in detail, in a language that I comprehend and I have fully understood the contents. I confirm that I have had opportunity to ask questions.

The nature and purpose of study and its potential risks/ benefits and expected duration of study, and relevant details of study have been explained to me in detail. I understand that my participation is voluntary and that I am free to withdraw at any time without giving any reasons, without my medical care or legal rights being affected.

I understand that the information collected about me from my participation in this research and sections of any of my medical notes may be looked at by responsible individuals from AIIMS. I give permission for these individuals to have access to my records.

I agree to take part in the above study.

-------------------------------------------

**(Signatures / Left Thumb Impression)**

Date: Name of the Participant: _________________________

Place: Son / Daughter / Spouse of:_______________________

Complete postal address: _________________________

**This is to certify that above consent have been obtained in my presence.**

Date : _______

Place: ________

-------------------------------------------

**(Signature of Principal Investigator)**

**Witness 1**

Name of the Witness ______________

Postal Address: _________________

Tel:____________________

**Witness 2**

Name of the Witness:______________

Postal Address: _________________

Tel: _________________________

# Appendix III

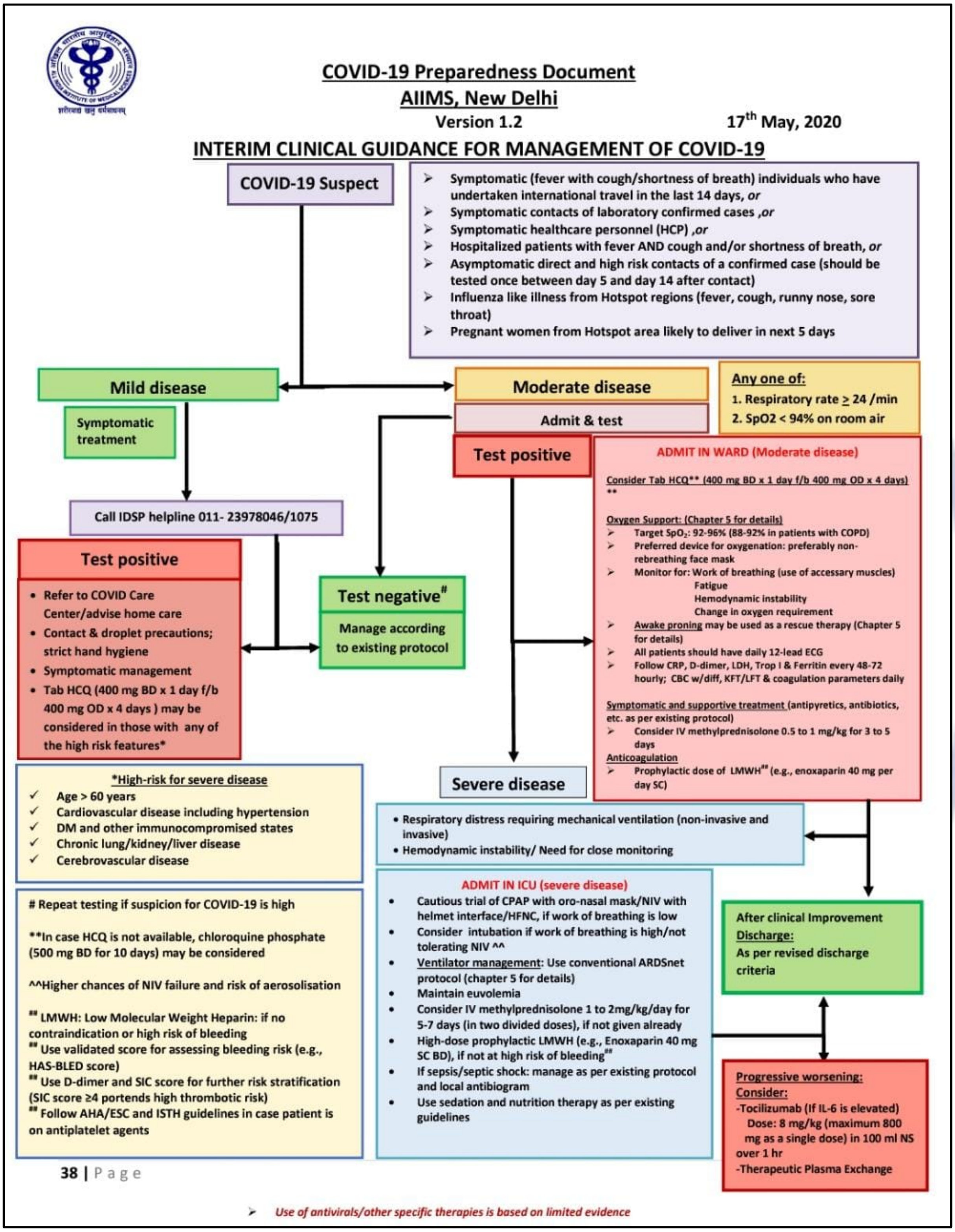

Supplement: Supplementary file 1 — Additional file 1. Full Study Protocol. [file 13063_2020_4840_MOESM1_ESM.pdf]
